# Aortic Arch Calcification Predicts the Renal Function Progression in Patients with Stage 3 to 5 Chronic Kidney Disease

**DOI:** 10.1155/2015/131263

**Published:** 2015-01-28

**Authors:** Lung-Chih Li, Yueh-Ting Lee, Yi-Wei Lee, Chia-An Chou, Chien-Te Lee

**Affiliations:** ^1^Division of Nephrology, Department of Internal Medicine, Kaohsiung Chang Gung Memorial Hospital and Chang Gung University College of Medicine, 123 Ta-Pei Road, Niao-Sung District, Kaohsiung 833, Taiwan; ^2^Department of Biomedical Imaging and Radiological Sciences, National Yang Ming University, Taipei 102, Taiwan; ^3^Department of Radiology, Kaohsiung Chang Gung Memorial Hospital and Chang Gung University College of Medicine, Kaohsiung 833, Taiwan

## Abstract

*Introduction*. The presence of aortic arch calcification (AoAC) and cardiomegaly on chest radiography has been demonstrated as important risk factors for cardiovascular mortality in patients with chronic kidney disease (CKD). However, the interrelationship among AoAC, cardiomegaly, and renal function progression remains unclear. The aim of this study is to assess whether AoAC and cardiomegaly are independently associated with the renal function progression in patients with stages 3–5 CKD. *Methods*. We retrospectively determined AoAC and cardiomegaly by chest X-ray in 237 patients, followed up for at least three years without entering dialysis and classified into 4 groups according to the presence or absence of AoAC and cardiomegaly. The change in renal function was measured by the slope of estimated glomerular filtration rate (eGFR). *Results*. Of the 237 patients, the rate of eGFR decline was significantly higher in the group with coexistence of AoAC and cardiomegaly than any other groups. Baseline AoAC and proteinuria were independently associated with eGFR decline. AoAC were independently determined by age, eGFR slope, and cardiomegaly. *Conclusions*. The coexistence of AoAC and cardiomegaly is associated with faster eGFR decline. AoAC is an independent determinant of renal outcomes in patients with CKD stages 3–5.

## 1. Introduction

Cardiovascular disease is the major cause of mortality in patients with all stages of chronic kidney disease (CKD) [1]. Several well-known traditional risk factors [2], such as old age, hypertension, diabetes, cigarette smoking, and dyslipidemia, have been found to partially, yet not fully, explain the high prevalence of cardiovascular events in these patients. Recently, the roles of nontraditional risk factors have been more emphasized [3]. Of these factors, vascular calcification and cardiomegaly have emerged as new potential risk factors for predicting the cardiovascular events in CKD patients [4, 5].

Vascular calcification is highly prevalent in patients with CKD compared to general population. More than half of CKD patients even before the start of dialysis and up to 80–90% of ESRD patients have various degrees of vascular calcification [6, 7]. Recently, aortic arch calcification (AoAC) on chest X-ray, which is an inexpensive and noninvasive exam, has been found to reflect the magnitude of whole aortic calcification in general population and CKD patients [8]. We and others have shown that AoAC is independently associated with cardiovascular and all-cause mortality in both CKD and ESRD patients [9–11]. However, the relationship of presence of AoAC and renal function progression remains unclear.

Increased cardiothoracic ratio (CTR) (>50%), another easily available parameter on chest radiography, is a marker of cardiomegaly and is considered predictive of left ventricular systolic dysfunction in chronic heart failure patients [12]. High CTR independently correlates with increased left ventricular mass and target organ damage in hypertensive patients [13]. Recently, increased CTR is shown to be correlated with malnutrition and inflammation and is predictive of two-year all-cause mortality in ESRD patients with hemodialysis and peritoneal dialysis [5, 14]. However, whether the CTR can predict the renal function deterioration in CKD patients remains unknown.

The aim of this study is to evaluate whether the presence of AoAC and/or cardiomegaly on chest radiographs can predict the renal function progression in patients with stages 3–5 CKD. We hypothesized that there is a possible interrelationship between AoAC and/or cardiomegaly on chest radiographs and progression of renal function so that it may be a useful tool for anticipation of renal outcome and for early intervention in these patients.

## 2. Subjects and Methods

### 2.1. Ethics Statement

This study was carried out in accordance with the Declaration of Helsinki and approved by the Institution Review Board of Kaohsiung Chang-Gung Memorial Hospital (IRB102-3070B).

### 2.2. Study Patients and Design

All CKD patients over 18 years of age were reviewed from the outpatients of Nephrology Department of Kaohsiung Chang-Gung Memorial Hospital between September 2006 and December 2012 in this retrospective observational study. We screened 598 predialysis patients with stage 3 to 5 CKD according to the National Kidney Foundation-Kidney Disease Outcomes Quality (KDOQI) guidelines [15]. Among them, a total of 269 patients who have been followed up for at least 3 years without entering end stage kidney disease or mortality were included in this study. We classified our patients with evidence of kidney injury lasting for more than 3 months into CKD stage 3, stage 4, and stage 5, based on estimated glomerular filtration rate (eGFR) level of 30 to 59, 15 to 29, and <15, respectively. Eighteen patients with less than three eGFR measurements during the follow-up period were excluded. Four patients who lack chest X-ray and 10 patients who had acute renal failure, defined by increase of creatinine up to 2 times or decrease of eGFR up to 50% in one month, were also excluded. Finally, 237 patients were enrolled in this study. Later the study patients were stratified into 4 groups according to whether they have AoAC with the absence or presence of cardiomegaly on chest X-rays.

### 2.3. Demographic and Clinical Data Collection

Demographic and medical data including age, gender, and comorbid conditions were obtained from medical records or interviews with patients. Body mass index (BMI) was calculated as weight/height^2^ (kg/m^2^). Blood was drawn after over 8 hours overnight fasting and the following laboratory data were measured from blood samples: hemoglobin, creatinine, blood urea nitrogen, calcium, phosphorus, sodium, potassium, albumin, total cholesterol, triglyceride, low density lipoprotein (LDL) cholesterol, and high density lipoprotein (HDL) cholesterol. Mean values of two measurements within the three months before or after chest radiography were used for analysis. The value of eGFR was calculated based on the simplified equation in the Modification of Diet in Renal Disease (MDRD) study [16], which is eGFR mL/min/1.73 m^2^ = 175 × serum creatinine^−1.154^  × age^−0.203^  × 0.742 (if female). Urine total protein to creatinine ratio (UPCR) was calculated as urine total protein (mg)/urine creatinine (g). The blood and urine sample were obtained on the same day.

### 2.4. Evaluation of AoAC and Cardiomegaly by Chest X-Ray

One experienced radiologist and one trained medical doctor blinded to the patients' clinical data independently reviewed the posterior-anterior (P-A) chest plain films of those CKD patients taken at the beginning of entering CKD care program. Calcification of aortic arch was assessed using a scale developed by Ogawa et al. [17]. The scale, dividing aortic arch into 16 sections by circumference, was attached to the aortic arch on chest X-rays and the number of sectors with calcification was counted. A calcification score of 2 and above was regarded as presence of calcification while a calcification score of 0 and 1 was regarded as absence of calcification. Cardiomegaly was defined as a transverse diameter of the cardiac silhouette greater than or equal to 50% of the transverse diameter of the chest on the P-A chest radiograph. To examine the intrareader variability, randomly selected 50 chest X-rays were examined by both readers. Five X-rays were scored differently on calcification score with a maximum difference of score ±2; two X-rays were judged differently on increased CTR due to borderline cardiomegaly. The discrepancies between the two observers were resolved by a third independent reader.

### 2.5. Statistical Analysis

Statistics analysis was performed using SPSS for windows version 18.0 (SPSS Inc., Chicago, IL, USA). Continuous variables were expressed as mean ± SD, and categorical variables were expressed as a number (percentage). Multiple comparisons among the four study groups were conducted by one-way analysis of variance (ANOVA) followed by post hoc test adjusted with Bonferroni's correction. Linear regression analysis was used to identify the factors associated with decline in kidney function and logistic regression analysis was used to determine the factors related to aortic calcification. Significant variables in univariate analysis were selected for multivariate analysis. A difference was considered significant if the *P* value was less than 0.05.

## 3. Results

The comparison of the baseline characteristics among the four study groups is demonstrated in Table 1. A total of 237 patients with stage 3 to 5 CKD were recruited. The mean age was 67.6 ± 13.2 years. The average age was oldest in the group with coexistence of AoAC and cardiomegaly (72.6 ± 11.6 years old), followed by AoAC only (71.1 ± 10.0 years old), cardiomegaly only (60.8 ± 13.7 years old), and none of each (60.2 ± 13.2 years old). Patients with coexistence of AoAC and cardiomegaly had the highest BMI (26.5 ± 5.0 kg/m^2^), the highest proportion of diabetes (58%), and the highest UPCR (1908.4 ± 2714.6 mg/g) among the four groups. Albumin was the lowest in patients with coexistence of AoAC and cardiomegaly (3.9 ± 0.6 mg/dL), while it was the highest in patients without AoAC or cardiomegaly (4.2 ± 0.5 mg/dL). LDL cholesterol was the highest among patients without AoAC or cardiomegaly (134.7 ± 81.2 mg/dL) whereas patients with cardiomegaly only had the lowest levels of LDL (95.7 ± 26.7). There was no significant difference in gender, hemoglobin, creatinine, eGFR, sodium, potassium, calcium, phosphorus, total cholesterol, triglyceride, or HDL among four groups.

### 3.1. Risks of Renal Function Decline

**Figure 1:**
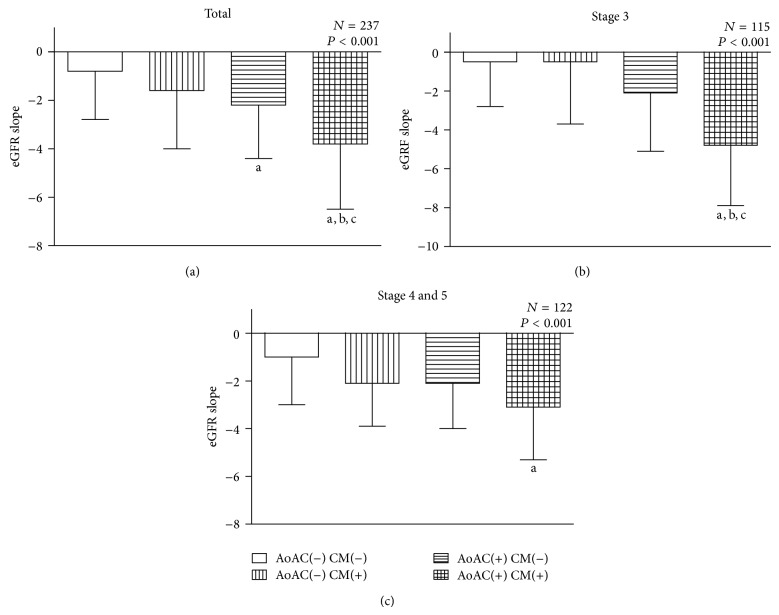
The eGFR slopes among 4 study groups: (a) in total 237 patients; (b) in patients with stage 3 CKD; (c) in patients with stages 4 and 5 CKD. ^a^ *P* < 0.05 compared with the group without AoAC nor CM. ^b^ *P* < 0.05 compared with the group with CM only. ^c^ *P* < 0.05 compared with the group with AoAC only. eGFR, estimated glomerular filtration; AoAC, aortic arch calcification; CM, cardiomegaly.

Figure 1 illustrates the eGFR slope in (a) total, (b) stage 3, and (c) stages 4 and 5 CKD patients. In all patients, the eGFR slopes in 4 groups were −0.8 ± 2.2, −1.6 ± 2.4, −2.2 ± 2.4, and −3.8 ± 2.7 mL/min/1.73 m^2^, respectively. The slope of eGFR decline was significantly higher in patients with coexistence of AoAC and cardiomegaly than all other groups. Patients with AoAC only had faster eGFR deterioration than patients without AoAC or cardiomegaly.

In stage 3 CKD patients, the eGFR slopes in 4 groups were −0.5 ± 2.3, −0.5 ± 3.2, −2.1 ± 3.4, and −4.8 ± 3.1 mL/min/1.73 m^2^, respectively. The group with coexistence of AoAC and cardiomegaly had a significantly greater eGFR decline than all other groups. However, in patients with stages 4 and 5 CKD, patients with coexistence of AoAC and cardiomegaly had only significantly greater eGFR decline than patients without AoAC or cardiomegaly (−3.1 ± 2.2 versus −1.0 ± 2.0 mL/min/1.73 m^2^, *P* < 0.01).

Table 2 shows the determinants of eGFR slope in all patients. Greater renal function progression had larger negative slope. In the univariate analysis, the eGFR slope had a significant positive correlation with albumin and negative correlation with age, diabetes, BMI, UPCR, baseline eGFR, AoAC, and cardiomegaly. In the multivariate analysis, the eGFR slope was correlated independently with UPCR (*β* = −0.203, *P* = 0.012) and AoAC (*β* = −0.224, *P* = 0.007), indicating that patients with more proteinuria and higher AoAC had faster progression of renal function.

### 3.2. Determinants of Aortic Arch Calcification

The determinants of AoAC are demonstrated in Table 3. The univariate regression analysis showed that old age, high BMI, presence of diabetes, cardiomegaly, and lower eGFR slope were associated with AoAC. In the multivariate study, AoAC was independently correlated with old age (odds ratio: 1.080, *P* < 0.0001), cardiomegaly (odds ratio: 2.491, *P* = 0.01), and a lower eGFR slope (odds ratio: 0.813, *P* < 0.0001).

## 4. Discussion

In the present study for a cohort of participants with stages 3–5 CKD, we found that both AoAC and cardiomegaly on chest X-ray are associated with renal function progression. AoAC is independently associated with renal function decline after adjustments for various covariates, suggesting that AoAC is not only a consequence of CKD but itself is predictive of renal function progression.

Multiple factors are identified and involved during the progression of CKD. There are modifiable factors such as hypertension and hyperglycemia. Adequate intervention can retard the decline of renal function and reduce cardiovascular mortality. It has been well recognized that detailed evaluation and appropriate management of risk factors are effective strategies for the treatment of CKD.

Calcification of aortic arch is not uncommon in general elderly population. It has been reported that 20–30% of people older than 65 years have calcification in the aorta [8]. In CKD patients, the prevalence of AoAC is even higher. In our cohort, more than half (60%) of the patients had various degrees of AoAC and, in agreement with previous studies [18], patients with AoAC are older than patients without AoAC. However, after adjusting the factor of age, AoAC still independently predicts the renal function deterioration in our study cohort.

AoAC may represent part of the vascular calcification in the whole body. A number of studies have shown an interrelationship between vascular calcification and increased arterial stiffness. Arterial stiffness, resulting from vascular calcification, is a powerful predictor of poor cardiovascular outcome [7]. Increased pulse pressure and pulse wave velocity, two indicators of arterial stiffness, were reported to be risk factors of rapid decline of renal function in CKD patients [19, 20] and in patients with relative normal baseline renal function [21, 22]. Although the mechanisms by which arterial stiffness causes renal damage remains incompletely understood, it has been proposed that the microcirculation, in which glomerular capillaries are positioned between afferent and efferent arterioles, increases susceptibility to injury under long-term increased pulse pressure [23]. These may explain, at least in part, that AoAC is associated with rapid renal function decline.

Of note, patients with coexistence of AoAC and increased CTR (cardiomegaly) are associated with greater renal function decline compared with patients with either AoAC or cardiomegaly or none of each. The eGFR decline of patients with coexistence of AoAC and cardiomegaly in stage 3, but not in later stages (stages 4 and 5), is close to rapid progression of renal function, defined by a decline in eGFR of more than 5 mL/min/1.73 m^2^ per year by K-DIQO guideline [24], implicating that presence of AoAC and cardiomegaly in the early stage of CKD may be associated with worse renal outcome. Although increased CTR is not an independent determinant of rapid renal function decline, AoAC is correlated with cardiomegaly in the current study. It has been shown that aortic arch calcification volume measured by multidirector computed tomography is independently correlated with left ventricular diastolic dysfunction [25]. Vascular calcification was also proved to cause hemodynamic alternations such as reduced compliance of large arteries and automatic dysfunction in hemodialysis patients [26]. Moreover, pulse wave velocity, as a determinant of arterial stiffness, is highly associated with LV hypertrophy in hemodialysis patients [25, 27]. Cardiomegaly may develop with AoAC and correlate with renal function progression.

Proteinuria is another independent determinant of rapid renal function deterioration in the present study. Baseline proteinuria is known to be associated with the risk of CKD progression in both nondiabetic [28] and diabetic [29, 30] patients. Recently Chen et al. also reported that various degrees of proteinuria may have different impact on cardiovascular events and all-cause mortality [31]. However, the interrelationship between proteinuria and vascular calcification remains unclear. One recent study has shown that coronary artery calcification is associated with albuminuria in type 2 diabetic patients [32]; however, our study did not support the role of proteinuria in AoAC. Instead, age, eGFR slope, and increased CTR are the independent determinants of AoAC.

Several limitations need to be acknowledged. First, since patients with early entering dialysis (<3 years) were excluded, the effects of AoAC on renal function may be underestimated in patients with late stage CKD (e.g., stage 5). Second, the use of AoAC and increased CTR on chest X-ray are not as sensitive and specific as other modalities such as CT scanning for calcification and cardiac sonography for cardiomegaly. Third, it is a relatively small-sized study, which may not have strong power to show the significance in some way. In addition, due to its observational and retrospective design, it may be subject to potential confounding from missing variables. Nevertheless we were still able to demonstrate an independent relation between AoAC and renal function progression by using this noninvasive and nonexpansive modality.

## 5. Conclusion

This study presents for the first time that baseline AoAC measured on chest radiography and proteinuria are independently associated with faster eGFR decline in patient with stages 3–5 CKD. AoAC are determined by age, eGFR slope, and cardiomegaly but not by proteinuria. The evaluation of AoAC on chest radiography may represent a simple, cheap, and noninvasive method to predict the renal function progression in these patients.

## Figures and Tables

**Table 1:** Clinical features and biochemical data of patients among study groups.

	Total	AoAC(−) CM(−)	AoAC(−) CM(+)	AoAC(+) CM(−)	AoAC(+) CM(+)	*P* value
	(*N* = 237)	(*N* = 65)	(*N* = 26)	(*N* = 55)	(*N* = 91)	
Age (years)	67.6 ± 13.2	60.2 ± 13.2	60.8 ± 13.7	71.1 ± 10.0^ab^	72.6 ± 11.6^ab^	<0.0001
Gender, male (%)	148 (62)	46 (71)	15 (58)	36 (66)	51 (56)	0.264
BMI (kg/m^2^)	24.5 ± 4.3	23.7 ± 3.5	24.9 ± 3.8	23.4 ± 3.2	26.5 ± 5.0^ac^	<0.0001
Diabetes (%)	109 (46)	24 (37)	10 (39)	22 (40)	53 (58)	0.010
UPCR (mg/g)	1313.3 ± 2197.8	846.1 ± 1319.6	1600.2 ± 3129.3	887.9 ± 1387.2	1908.4 ± 2714.6^a^	0.028
Hemoglobin (mg/dL)	11.8 ± 2.4	12.2 ± 2.2	12.2 ± 2.8	11.6 ± 2.5	11.4 ± 2.4	0.149
Serum creatinine (mg/dL)	2.5 ± 1.2	2.3 ± 1.2	2.7 ± 1.6	2.7 ± 1.3	2.5 ± 1.1	0.207
eGFR (mL/min/1.73 m^2^)	30.3 ± 15.6	34.6 ± 15.6	31.1 ± 19.7	27.9 ± 14.9	29.3 ± 14.4	0.086
Sodium (meq/L)	140.3 ± 2.8	140.8 ± 2.1	140.5 ± 2.6	140.3 ± 2.6	140.0 ± 3.2	0.397
Potassium (meq/L)	4.4 ± 0.6	4.4 ± 0.6	4.3 ± 0.6	4.4 ± 0.5	4.5 ± 0.6	0.805
Calcium (mg/dL)	8.9 ± 0.5	8.9 ± 0.5	9.0 ± 0.5	8.8 ± 0.4	8.9 ± 0.6	0.584
Phosphorus (mg/dL)	3.7 ± 0.8	3.6 ± 0.9	3.7 ± 0.9	3.7 ± 0.7	3.7 ± 0.8	0.897
Ca × P	32.2 ± 8.4	32.2 ± 8.7	32.7 ± 8.0	32.6 ± 5.8	31.7 ± 9.6	0.935
Albumin (mg/dL)	4.1 ± 0.5	4.2 ± 0.5	4.1 ± 0.5	4.2 ± 0.4	3.9 ± 0.6^ac^	0.004
Total cholesterol (mg/dL)	194.8 ± 61.7	209.9 ± 89.2	181.4 ± 30.4	190.7 ± 45.2	190.6 ± 52.5	0.186
Triglyceride (mg/dL)	163.4 ± 118.5	164.1 ± 118.2	190.6 ± 121.9	150.8 ± 132.7	163.8 ± 108.7	0.637
HDL	48.2 ± 15.3	49.7 ± 16.4	46.8 ± 15.1	51.9 ± 14.7	45.1 ± 14.4	0.124
LDL	115.8 ± 53.9	134.7 ± 81.2	95.7 ± 26.7^a^	114.2 ± 34.8	108.7 ± 39.9	0.023

BMI: body mass index; UPCR: urine protein-creatinine ratio; eGFR: estimated glomerular filtration rate; HDL: high density lipoprotein cholesterol; LDL: low density lipoprotein cholesterol; AoAC: aortic arch calcification; CM: cardiomegaly; ^a^ *P* < 0.05 compared with AoAC(−) CM(−); ^b^ *P* < 0.05 compared with AoAC(−) CM(+); ^c^ *P* < 0.05 compared with AoAC(+) CM(−).

**Table 2:** Determinants associated with eGFR slope in study patients (linear regression).

	Univariate		Multivariate	
	Standardized coefficient *β*	*P* value	Standardized coefficient *β*	*P* value
Age (per 1-year increase)	−0.152	0.015	−0.103	0.201
Male gender	0.034	0.593	—	—
Diabetes	−0.235	<0.0001	−0.102	0.176
BMI (per 1 m^2^ increase)	−0.194	0.003	−0.118	0.124
UPCR (mg/g)	−5.729	<0.0001	−0.203	0.012
Baseline eGFR	−0.010	0.037	−0.049	0.518
Calcium	−0.051	0.446	—	—
Phosphorus	0.039	0.564	—	—
Ca × P	0.009	0.890	—	—
Albumin	0.133	0.047	0.100	0.211
Total cholesterol (mg/dL)	0.093	0.175	—	—
Triglyceride (mg/dL)	−0.070	0.306	—	—
HDL	−0.003	0.969	—	—
LDL	0.145	0.051	—	—
AoAC	−0.253	<0.0001	−0.224	0.007
Cardiomegaly	−0.131	<0.0001	−0.039	0.620

BMI: body mass index; UPCR: urine protein-creatinine ratio; eGFR: estimated glomerular filtration rate; HDL: high density lipoprotein cholesterol; LDL: low density lipoprotein cholesterol, AoAC: aortic arch calcification.

**Table 3:** Determinants of aortic arch calcification in study patients (logistic regression).

	Univariate		Multivariate	
	Odds ratio (95% CI)	*P* value	Odds ratio (95% CI)	*P* value
Age (per 1-year increase)	1.085 (1.058–1.114)	<0.0001	1.080 (1.048−1.111)	<0.0001
Male gender	0.737 (0.438–1.241)	0.252	—	—
Diabetes	1.533 (1.045–2.904)	0.001	0.821 (0.426−1.595)	0.575
BMI (per 1 m^2^ increase)	1.071 (1.002–1.145)	0.042	0.993 (0.923−1.085)	0.875
UPCR (mg/g)	1.319 (0.741–2.346)	0.347	—	—
Baseline eGFR	0.989 (0.973–1.004)	0.144	—	—
CKD stage	1.196 (0.868–1.650)	0.274	—	—
eGFR slope	0.842 (0.777–0.911)	<0.0001	0.813 (0.773−0.938)	<0.0001
Calcium	1.036 (0.648–1.657)	0.882	—	—
Phosphorus	0.910 (0.676–1.226)	0.536	—	—
Ca × P	0.982 (0.951–1.013)	0.251	—	—
Albumin	0.770 (0.464–1.276)	0.310	—	—
Total cholesterol (mg/dL)	0.997 (0.993–1.002)	0.273	—	—
Triglyceride (mg/dL)	0.999 (0.997–1.002)	0.567	—	—
HDL	0.999 (0.980–1.019)	0.952	—	—
LDL	0.996 (0.989–1.002)	0.166	—	—
Cardiomegaly	3.911 (2.289–6.683)	<0.0001	2.491 (1.326−4.996)	0.010

BMI: body mass index; UPCR: urine protein-creatinine ratio; CKD: chronic kidney disease; eGFR: estimated glomerular filtration rate; HDL: high density lipoprotein cholesterol; LDL: low density lipoprotein cholesterol.
